# Materialism Profiles Across Levels of Subjective Family Economic Status and Their Prospective Associations with Subjective Well-Being Among Chinese Vocational College Students

**DOI:** 10.3390/bs16091511

**Published:** 2026-08-27

**Authors:** Zhengqi Wei, Cheng Guo

**Affiliations:** 1 Faculty of Psychology, Southwest University, Chongqing 400715, China; ellawei@email.swu.edu.cn; 2 Office of Academic Affairs and Faculty of Teacher Education, West Yunnan University, Lincang 677000, China; 3 Yibin Academy of Southwest University, Yibin 644000, China

**Keywords:** materialism, subjective family economic status, subjective well-being, latent profile analysis, vocational college students

## Abstract

Materialistic values and family economic circumstances have both been linked to well-being, but little is known about whether materialism profiles are similar across family economic contexts and how they relate to subsequent well-being. A sample of 1641 Chinese higher vocational college students completed two surveys six months apart. Materialism and subjective family economic status (SFES) were assessed at T1, whereas life satisfaction and depressive symptoms were assessed at T1 and T2. Separate latent profile analyses conducted within each of the three SFES groups consistently yielded low-, moderate-, and high-materialism profiles, and multiple-group analyses further supported similarity in profile means, indicator variances, and profile proportions across SFES levels. BCH analyses adjusting for the corresponding T1 outcome and demographic covariates showed significant overall variation in T2 outcomes across combinations of SFES group and materialism profile. Across SFES groups, students in the high-materialism profile had significantly higher depressive symptoms than those in the low-materialism profile after Holm correction, whereas corresponding life-satisfaction differences were not significant. Interaction-type tests showed no significant differences across SFES groups in the associations between materialism profiles and either well-being outcome. Overall, materialism profiles were associated with subsequent well-being, particularly depressive symptoms, across perceived family economic circumstances.

## 1. Introduction

Subjective well-being is a central indicator of young adults’ psychological adjustment, reflecting both positive evaluations of life and the relative absence of psychological distress (Busseri & Sadava, 2011; Diener, 1984; Suldo & Shaffer, 2008). Consistent with this multidimensional view, life satisfaction and depressive symptoms have been used to capture the positive and negative aspects of young adults’ well-being (Diener et al., 1985; Ong et al., 2021). High levels of subjective well-being are associated with adaptive developmental outcomes, whereas low well-being may increase vulnerability to internalizing problems and emotional distress (Keyes, 2005; Lyubomirsky et al., 2005). These concerns warrant particular attention in vocational education contexts. Prior research has identified subjective well-being as an important concern among vocational students (Yang & Yu, 2024; Yuan et al., 2021), especially in the Chinese cultural and educational context, where academic achievement and educational credentials are strongly emphasized (Tang & Nie, 2023). In China, students in vocational education may encounter a range of developmental and contextual challenges, including concerns about educational status, social recognition, future career opportunities, and transitions into the labor market (Tang & Nie, 2023; G. Wang & Wang, 2023). These experiences may negatively affect vocational students’ perceptions of themselves and their future prospects, which may in turn be associated with lower life satisfaction and heightened depressive symptoms (He et al., 2023; Zeng et al., 2022). Therefore, it is important to further examine factors associated with subjective well-being among vocational college students.

Previous research has examined a wide range of factors associated with psychological adjustment and well-being among adolescents and young adults, including school-related factors such as school climate (M.-T. Wang & Degol, 2016), peer relationships (van Harmelen et al., 2017), and academic pressure (Steare et al., 2023), as well as family-related factors such as parental support and parent–child relationships (Wu & Lee, 2022; Zhu & Shek, 2021) and individual factors such as self-esteem (Orth et al., 2012). Although this literature has substantially advanced understanding of young people’s well-being, value orientations may provide an additional perspective for understanding psychological adjustment, particularly materialistic values. Materialistic values may be relevant in vocational education contexts, where concerns about future income, occupational development, and socioeconomic mobility can be salient as students prepare for the transition from education to employment (G. Wang & Wang, 2023). Moreover, the social recognition and perceived status associated with vocational education may shape how students evaluate achievement and future success (Tang & Nie, 2023). As a result, material success may come to represent not only a desirable life outcome but also a symbolic marker of personal achievement, social recognition, and future socioeconomic mobility.

Materialism refers to the importance individuals attach to wealth, possessions, and material acquisition in defining success, organizing their lives, and pursuing happiness (Richins & Dawson, 1992). Accordingly, materialism has been conceptualized as comprising three related dimensions: Success, reflecting the use of possessions as indicators of achievement; Centrality, reflecting the extent to which acquisition occupies a central place in life; and Happiness, reflecting the belief that possessions and acquisition are important sources of happiness (Richins & Dawson, 1992; Richins, 2004). From the perspective of self-determination theory (SDT), materialistic values represent an orientation toward extrinsic goals, such as wealth, status, and image, relative to intrinsic goals such as personal growth, close relationships, and community contribution (Deci & Ryan, 2000; Kasser, 2016). Because intrinsic rather than extrinsic goal pursuits are generally more conducive to the satisfaction of basic psychological needs for autonomy, competence, and relatedness, SDT provides a theoretical basis for expecting stronger materialistic orientations to be associated with poorer psychological adjustment (Ryan & Deci, 2017). Consistent with this perspective, a large body of research has linked stronger materialistic values to poorer subjective well-being, including lower life satisfaction and higher levels of depression and anxiety (Dittmar et al., 2014; Kasser, 2016). However, much of the existing evidence is cross-sectional and has focused on adolescents or general college student populations. Although longitudinal evidence has emerged among general university students and working adults (Reyes et al., 2024; R. Wang et al., 2017), prospective evidence among vocational college students remains comparatively limited.

Subjective family economic status (SFES) is another contextual factor that may be relevant to vocational students’ well-being (Yan et al., 2022). It reflects individuals’ subjective perceptions of their family’s economic circumstances (Davisson et al., 2025). Students who perceive their family economic circumstances as less favorable may experience greater concern about their economic prospects and fewer perceived opportunities, whereas more favorable perceptions may be associated with a greater sense of economic security and access to resources. Previous research among Chinese students has shown that less favorable perceptions of family economic circumstances are associated with poorer psychological adjustment and well-being (Dong et al., 2023; Yan et al., 2022). Scarcity theory offers one perspective for understanding why economic constraints may be relevant to psychological well-being (Mullainathan & Shafir, 2013; Shah et al., 2012). According to this theory, perceived insufficiency of important resources can heighten the salience of resource-related concerns, directing greater attention toward economic needs and constraints. From this perspective, students who perceive greater constraints in their family economic circumstances may be more attentive to financial resources and future economic opportunities, which may in turn be relevant to their well-being.

Materialistic values and SFES capture two distinct but potentially interrelated aspects of students’ experiences: materialism reflects a value orientation centered on material success and acquisition, whereas SFES reflects students’ perceived family economic circumstances. Self-determination theory and scarcity theory provide complementary perspectives for considering these two aspects together. SDT emphasizes the potential well-being implications of prioritizing extrinsic material goals (Deci & Ryan, 2000; Kasser, 2016), whereas scarcity theory highlights the psychological relevance of perceived economic constraints (Mullainathan & Shafir, 2013; Shah et al., 2012). Taken together, these perspectives suggest that family economic circumstances may provide a meaningful context for understanding materialistic values and their associations with well-being. The psychological significance of materialistic values may therefore vary across different family economic contexts, as material goals may be experienced differently depending on perceived resources and opportunities. This possibility raises the question of whether patterns of materialism are similar across different levels of SFES and whether their associations with subsequent well-being are consistent across these economic contexts.

A person-centered approach is particularly useful for examining heterogeneity in materialistic values across different family economic contexts. Rather than focusing solely on average associations among variables, latent profile analysis allows researchers to identify subgroups of individuals who show similar patterns of materialistic values, thereby capturing individual differences that may not be fully reflected by an overall score (Morin et al., 2016). Applying this approach across different levels of SFES makes it possible to examine what materialism profiles emerge within each economic context, whether the structure and prevalence of these profiles are similar across SFES groups, and whether their associations with subsequent life satisfaction and depressive symptoms are consistent across different family economic backgrounds. In this way, a person-centered approach can complement variable-centered analyses by providing a more differentiated understanding of the interplay among materialism, family economic context, and subsequent well-being.

### The Current Study

Although materialistic values and family economic circumstances have both been linked to well-being, less is known about the heterogeneity of materialism across different family economic contexts and whether its associations with well-being are consistent across these contexts. Much of the existing research has relied on variable-centered approaches (e.g., Flurry et al., 2021; R. Wang et al., 2017), which are well suited to examining average associations among variables but provide less information about distinct patterns of materialistic values within individuals. A person-centered approach can therefore provide a complementary perspective by identifying heterogeneity in materialism and examining whether similar profiles and well-being correlates emerge across different levels of subjective family economic status (SFES).

Guided by self-determination theory and informed by scarcity theory, the present study used a two-wave prospective design to examine materialism profiles across different levels of SFES among Chinese higher vocational college students. Specifically, we first identified the materialism profiles that emerged within each SFES group (Research Question 1). We then examined the extent to which the structure and prevalence of these profiles were similar across different family economic contexts (Research Question 2). Finally, we examined whether the materialism profiles were associated with life satisfaction and depressive symptoms six months later, after accounting for the corresponding baseline well-being indicator and relevant demographic covariates, and whether these profile–well-being associations were consistent across SFES groups or varied according to family economic circumstances (Research Question 3). Complementary variable-centered analyses were also conducted to examine whether the continuous associations between materialism and subsequent well-being varied across levels of SFES.

## 2. Materials and Methods

### 2.1. Participants and Procedure

The baseline analytic sample comprised 4012 students from five higher education institutions in Southwest China. The institutions were recruited on the basis of accessibility and existing academic collaborations with the research team. T1 data were collected in October during the fall semester, and T2 data were collected approximately six months later, in April of the following spring semester. Three of the five institutions participated in the T2 follow-up. The remaining two institutions, which had contributed 166 participants at baseline, did not continue to the T2 follow-up owing to limited institutional willingness to participate in the follow-up. Accordingly, 3846 baseline participants from the three continuing institutions were eligible for individual-level follow-up.

Questionnaires were administered online and distributed to students by the participating institutions. Participation was voluntary, and no monetary or non-monetary incentives were provided. Students were informed of the study purpose, procedures, confidentiality protections, and their right to discontinue participation. Records across waves were matched using participants’ names and student identification numbers. Of the 3846 baseline participants eligible for individual-level follow-up, 1641 were successfully matched across T1 and T2, corresponding to a retention rate of 42.7% among those eligible for follow-up. Relative to the full T1 baseline sample of 4012 participants, the overall T1–T2 matched proportion was 40.9%. Participants who did not complete T2 or could not be matched across waves were treated as not retained. Individual reasons for nonparticipation at T2 were not systematically recorded.

The matched analytic sample comprised 1641 students, of whom 69.8% were female. Participants ranged in age from 16 to 24 years (*M* = 18.68, *SD* = 0.87). First-, second-, and third-year students accounted for 72.3%, 26.1%, and 1.6% of the sample, respectively. In addition, 65.6% came from rural areas, 20.2% were only children, and 49.3% were left-behind students (i.e., students whose parents were currently working away from home for more than three months, living apart from one or both parents at the time of the survey). Participants represented a broad range of study programs, including business and financial management, logistics and transportation, electronic and communication engineering, computing and artificial intelligence, nursing and rehabilitation, public and social services, digital media and arts, funeral services, and eldercare.

This study was approved by the Ethics Committee of the Faculty of Psychology at Southwest University of China (IRB protocol number: SWU-ECHR-20250241). Permission was obtained from the participating schools, and informed consent was obtained from all participants. For participants under 18 years of age, informed consent was additionally obtained from their parents or legal guardians before data collection. Participants were assured of the confidentiality of their personal information and responses. Furthermore, every effort was made to ensure that no physical or psychological harm would be caused to the participants.

### 2.2. Measures

#### 2.2.1. Materialistic Values

Materialistic values were assessed at T1 using the 13-item Material Values Scale (Li & Guo, 2009), adapted from Richins and Dawson (1992). The scale comprises three dimensions: Success (5 items), Centrality (5 items), and Happiness (3 items). Sample items include “Acquiring material possessions is one of the most important achievements in life” (Success), “I enjoy spending money on things that are not practical” (Centrality), and “My life would be better if I owned certain things I do not have” (Happiness). Items were rated on a 5-point Likert scale ranging from 1 (strongly disagree) to 5 (strongly agree). Mean scores were computed, with higher scores indicating greater materialism. Reverse-keyed items were recoded before scale scores were calculated.

In the present analytic sample, Cronbach’s α was 0.75 for the total scale and 0.55, 0.53, and 0.49 for the Success, Centrality, and Happiness subscales, respectively. Similar subscale coefficients have been reported in a previous study of Chinese vocational students using the same scale (α = 0.56, 0.61, and 0.42, respectively; Song & Chen, 2019). As a supplementary reliability check in light of the reverse-keyed items, Spearman–Brown corrected split-half coefficients were calculated using a fixed item-splitting scheme that distributed reverse-keyed items across the two halves where possible; the coefficients were 0.67, 0.63, and 0.62 for Success, Centrality, and Happiness, respectively.

An item-level confirmatory factor analysis was conducted to examine the theoretically specified three-factor structure. Given the presence of both positively and reverse-keyed items, an orthogonal reverse-wording method factor defined by the five reverse-keyed items was included to account for wording-related method variance. The resulting model showed acceptable fit, *χ*^2^(52) = 287.28, CFI = 0.934, TLI = 0.901, RMSEA = 0.053, 90% CI [0.047, 0.059], and SRMR = 0.040, providing additional evidence broadly consistent with the theoretically specified three-dimensional structure. Given the modest subscale reliabilities, an item-level LPA sensitivity analysis was also conducted to examine whether the overall profile pattern depended on aggregation into the three subscale scores.

#### 2.2.2. Subjective Family Economic Status

Subjective family economic status (SFES) was measured at T1 using a single-item indicator of perceived family economic condition. Participants were asked, “What do you think of your family’s current economic condition?” Response options were coded as 0 (difficult), 1 (below average), 2 (average), and 3 (very comfortable), with higher scores indicating a more favorable perceived family economic condition. Conceptually similar adolescent-reported measures of subjective family economic circumstances have been used in prior research (Davisson et al., 2025; Hammond et al., 2021). These measures have shown meaningful associations with other indicators of family socioeconomic status, including parent-reported measures and, in more recent work, administrative indicators (Davisson et al., 2025; Hammond et al., 2021). In this study, SFES refers specifically to participants’ subjective assessment of their family’s economic circumstances and should not be interpreted as a comprehensive socioeconomic-status measure incorporating parental education, occupation, household income, or broader social position.

The four response categories included 335 participants (20.4%) in the difficult category, 610 (37.2%) in the below-average category, 686 (41.8%) in the average category, and 10 (0.6%) in the very-comfortable category. Because only 10 participants selected the highest category, the average and very-comfortable categories were combined for the person-centered analyses, resulting in three observed SFES groups: difficult (*n* = 335, 20.4%), below average (*n* = 610, 37.2%), and average or above (*n* = 696, 42.4%).

#### 2.2.3. Life Satisfaction

Life satisfaction was measured at T1 and T2 using three items adapted from the Satisfaction With Life Scale (Diener et al., 1985). A sample item is “I am satisfied with my life.” Participants rated each item on a 5-point Likert scale ranging from 1 (strongly disagree) to 5 (strongly agree). Mean scores were computed, with higher scores indicating greater life satisfaction. Cronbach’s α coefficients were 0.79 at T1 and 0.78 at T2.

Because a single-factor CFA with three indicators is just identified, conventional global fit indices are not informative for evaluating the three-item measure at an individual wave. Longitudinal measurement invariance was therefore examined across T1 and T2. Metric invariance was supported, and partial scalar invariance was obtained after allowing one item intercept to vary across waves *χ*^2^(8) = 7.37, *p* = 0.497, CFI = 1.000, TLI = 1.000, RMSEA = 0.000, and SRMR = 0.011.

#### 2.2.4. Depressive Symptoms

Depressive symptoms were assessed at T1 and T2 using the 9-item Patient Health Questionnaire (PHQ-9; Kroenke et al., 2001). Participants indicated how often they had experienced each symptom during the past two weeks on a 4-point scale ranging from 0 (not at all) to 3 (nearly every day). A sample item is “Feeling down, depressed, or hopeless.” Mean scores were computed across all items for the primary analyses, with higher mean scores indicating more severe depressive symptoms. Because the mean score is a linear rescaling of the conventional 0–27 total score, this scoring choice does not alter participants’ relative ordering. The PHQ-9 was analyzed as a continuous symptom measure and was not used for diagnostic classification.

Cronbach’s α coefficients were 0.88 at T1 and 0.89 at T2. At T2, a single-factor CFA showed acceptable fit, *χ*^2^(27) = 220.61, CFI = 0.939, TLI = 0.919, RMSEA = 0.066, 90% CI [0.058, 0.074], and SRMR = 0.040. Longitudinal analyses further supported metric invariance and partial scalar invariance across T1 and T2, *χ*^2^(140) = 619.73, CFI = 0.939, TLI = 0.933, RMSEA = 0.046, and SRMR = 0.040.

#### 2.2.5. Demographic Variables

Demographic variables included gender, age, grade, residence, only-child status, and left-behind status. Gender was coded as 0 = male and 1 = female; residence as 0 = urban and 1 = rural; grade as 1 = first year, 2 = second year, and 3 = third year; only-child status as 0 = no and 1 = yes; and left-behind status as 0 = no and 1 = yes. Age was treated as a continuous variable. Gender, age, residence, and only-child status were included as demographic covariates in the adjusted distal-outcome models; grade and left-behind status were included in descriptive, correlational, and attrition analyses.

### 2.3. Data Analysis

Analyses were conducted using IBM SPSS Statistics 27.0 and Mplus 8.3. Robust maximum likelihood estimation (MLR) was used for the latent profile models, BCH analyses with continuous distal outcomes, and variable-centered regression models. No item-level missing data were observed for the study measures in the matched analytic sample; therefore, no imputation was required.

First, attrition analyses compared participants from the three follow-up institutions who were retained and successfully matched at T2 (*n* = 1641) with those who were not retained or matched (*n* = 2205). Continuous baseline characteristics were compared using independent-samples *t* tests, with Welch’s unequal-variances *t* test used when the homogeneity-of-variance assumption was not met. Categorical variables were compared using chi-square tests. Cohen’s *d* and Cramér’s *V* were reported as effect-size indices.

Second, descriptive statistics, internal consistency coefficients, and Pearson correlations were calculated. Confirmatory factor analyses were conducted for the materialism and depressive-symptom measures, and longitudinal measurement invariance was evaluated for life satisfaction and depressive symptoms across T1 and T2. The distribution of PHQ-9 scores was also examined to evaluate the appropriateness of the primary continuous-outcome specification.

Third, person-centered analyses were conducted to examine heterogeneity in materialism within different levels of SFES. The Success, Centrality, and Happiness subscale scores were used as continuous LPA indicators. Because SFES was an ordinal single-item measure, it was treated as an observed grouping variable rather than as an LPA indicator. We first conducted separate LPAs within each of the three observed SFES groups (difficult, below average, and average or above). For each SFES group, one- through five-profile models were estimated and compared to determine the most appropriate number of materialism profiles. Indicator means were freely estimated across profiles, whereas indicator variances were constrained to equality across profiles and within-profile covariances were fixed to zero, consistent with a parsimonious LPA specification. Model selection considered the Akaike information criterion (AIC), Bayesian information criterion (BIC), sample-size-adjusted BIC (aBIC), entropy, adjusted Lo-Mendell-Rubin likelihood ratio test (aLMR), bootstrap likelihood ratio test (BLRT), profile size, substantive interpretability, and stability of the best log-likelihood (Nylund et al., 2007). Single-group enumeration models were estimated using 4000 initial-stage random starts and 1000 final-stage optimizations, and the best log-likelihood was replicated for all retained solutions.

Following identification of a common three-profile representation, cross-SFES profile similarity was evaluated sequentially (Morin et al., 2016). MG1 was a configural model in which profile means, indicator variances, and profile proportions were freely estimated across SFES groups; MG2 imposed equality constraints on profile means; MG3 additionally constrained indicator variances; and MG4 additionally constrained profile proportions. Model comparisons emphasized the BIC, aBIC, and consistent AIC (CAIC); a more constrained model was retained when at least two of these three information criteria decreased relative to the preceding model, together with consideration of entropy, convergence, parsimony, and substantive interpretability. The multiple-group models were estimated using 8000 initial-stage random starts and 2000 final-stage optimizations, and the retained model was re-estimated with 12,000 and 3000 starts, respectively, as a stability check.

For the distal-outcome analyses, the BCH procedure (Asparouhov & Muthén, 2014) was used to account for uncertainty in latent profile assignment. The corresponding T1 outcome and demographic covariates (gender, age, residence, and only-child status) were included as predictors of both latent profile membership and the T2 distal outcome. Thus, T1 life satisfaction was included in analyses of T2 life satisfaction, whereas T1 depressive symptoms were included in analyses of T2 depressive symptoms. Overall Wald tests evaluated differences across the nine combinations of SFES group and materialism profile, and interaction-type Wald tests examined whether materialism-profile differences varied across SFES groups. Three theoretically focused high-versus-low materialism contrasts were tested, one within each SFES group. Mean differences, standard errors, 95% confidence intervals, and standardized differences were reported. The Holm correction (Holm, 1979) was applied separately to the three planned contrasts within each outcome to control the family-wise error rate.

Finally, two sensitivity analyses and one complementary variable-centered analysis were conducted. First, because PHQ-9 scores were positively skewed and included a substantial proportion of zero scores, the BCH depressive-symptom analysis was repeated using the conventional 0–27 PHQ-9 total score under a negative binomial specification, with the same baseline and demographic adjustments as in the primary analysis. Second, given the modest internal consistency of the three materialism subscales, the LPA was repeated in the full analytic sample using all 13 individual materialism items directly as indicators, rather than using the three subscale scores. Third, to examine whether the substantive conclusions depended on the person-centered representation of materialism, complementary variable-centered regression models were estimated for each T2 outcome. Continuous T1 total materialism was grand-mean centered, the three-level SFES variable was represented by two dummy variables with the difficult group as the reference category, and Materialism × SFES interaction terms were included. These models used MLR, controlled for the corresponding T1 outcome and the same demographic covariates as the BCH analyses, and used a two-degree-of-freedom Wald test to evaluate the omnibus Materialism × SFES interaction.

## 3. Results

### 3.1. Attrition Analysis

Attrition analyses were restricted to participants from the three institutions that participated in both waves, excluding the 166 participants from institutions that did not participate at T2. The analyses compared the 1641 participants retained and successfully matched at T2 with the 2205 participants who were not retained or matched. Several demographic characteristics differed significantly between the two groups. Participants retained at T2 were younger than those not retained (*M* = 18.68 vs. 18.91 years), Welch’s *t*(3771.74) = 7.52, *p* < 0.001, *d* = 0.24. Gender composition also differed, *χ*^2^(1) = 72.00, *p* < 0.001, Cramér’s *V* = 0.14, and grade distribution differed, *χ*^2^(2) = 132.20, *p* < 0.001, Cramér’s *V* = 0.19. Only-child status showed a small difference, *χ*^2^(1) = 4.07, *p* = 0.044, Cramér’s *V* = 0.03. Among the substantive study variables, the retained group reported a slightly higher Centrality score than the nonretained group (*M* = 2.68 vs. 2.63), *t*(3844) = 2.46, *p* = 0.014, *d* = 0.08. No statistically significant differences were observed for total materialism, Success, Happiness, T1 life satisfaction, T1 depressive symptoms, SFES, residence, or left-behind status (all *ps* > 0.05). Overall, attrition was selective primarily with respect to age, gender, and grade, whereas differences in the principal psychological variables were small or nonsignificant. Age and gender were included in the adjusted outcome models; grade was not additionally included because of its substantial overlap with age.

### 3.2. Descriptive Statistics, Correlations, and Distributional Checks

Descriptive statistics and bivariate correlations are presented in Table 1. T1 materialism was negatively associated with life satisfaction and positively associated with depressive symptoms at both T1 and T2. Among the three materialism dimensions, Success showed the strongest associations with the well-being indicators. The corresponding well-being indicators were positively correlated across waves, with T1 life satisfaction associated with T2 life satisfaction and T1 depressive symptoms associated with T2 depressive symptoms. SFES was positively associated with life satisfaction and negatively associated with depressive symptoms at both time points. Demographic associations were generally small, apart from the expected positive association between age and grade.

The PHQ-9 distribution was positively skewed. At T2, the conventional 0–27 total score had a mean of 2.99 (*SD* = 3.50), a median of 2, and a skewness of 1.54; 32.7% of participants had a total score of zero. At T1, the corresponding values were *M* = 3.64, *SD* = 3.76, median = 3, and skewness = 1.44, with 25.4% scoring zero. These distributional characteristics motivated an additional negative binomial sensitivity analysis of depressive symptoms.

### 3.3. Materialism Profile Identification Within SFES Groups

Separate LPAs were estimated within each of the three observed SFES groups. Table 2 presents the fit statistics for one- through five-profile solutions. Across the three groups, the three-profile solutions provided the most defensible balance of statistical fit, profile size, parsimony, interpretability, and cross-group comparability. In the difficult SFES group, the three-profile solution had the lowest BIC and was supported by a significant aLMR test relative to the two-profile solution. Although AIC and aBIC continued to decrease in the four- and five-profile solutions, these models were not supported by the aLMR test and yielded very small profiles. In the below-average and average-or-above groups, higher-profile solutions yielded some improvement in statistical fit but introduced very small profiles and primarily further subdivided the same low-to-moderate-to-high materialism pattern. Accordingly, a common three-profile representation was retained across the three SFES groups.

For the retained three-profile solutions, average posterior probabilities for the assigned profiles ranged from 0.864 to 0.871 in the difficult group, from 0.822 to 0.896 in the below-average group, and from 0.848 to 0.892 in the average-or-above group. The best log-likelihood value was replicated across random starts for each retained solution.

### 3.4. Cross-SFES Profile Similarity and Model Selection

Four multiple-group models were compared to align substantively comparable materialism profiles across the three SFES groups (Table 3). Although AIC favored the less constrained MG1 model, BIC, aBIC, and CAIC decreased at each successive similarity step, while entropy remained essentially unchanged. Based on the information-criterion framework described above, MG4, which constrained profile means, indicator variances, and profile proportions to equality across SFES groups, was retained as the final multiple-group profile model for the subsequent BCH analyses.

**Figure 1:**
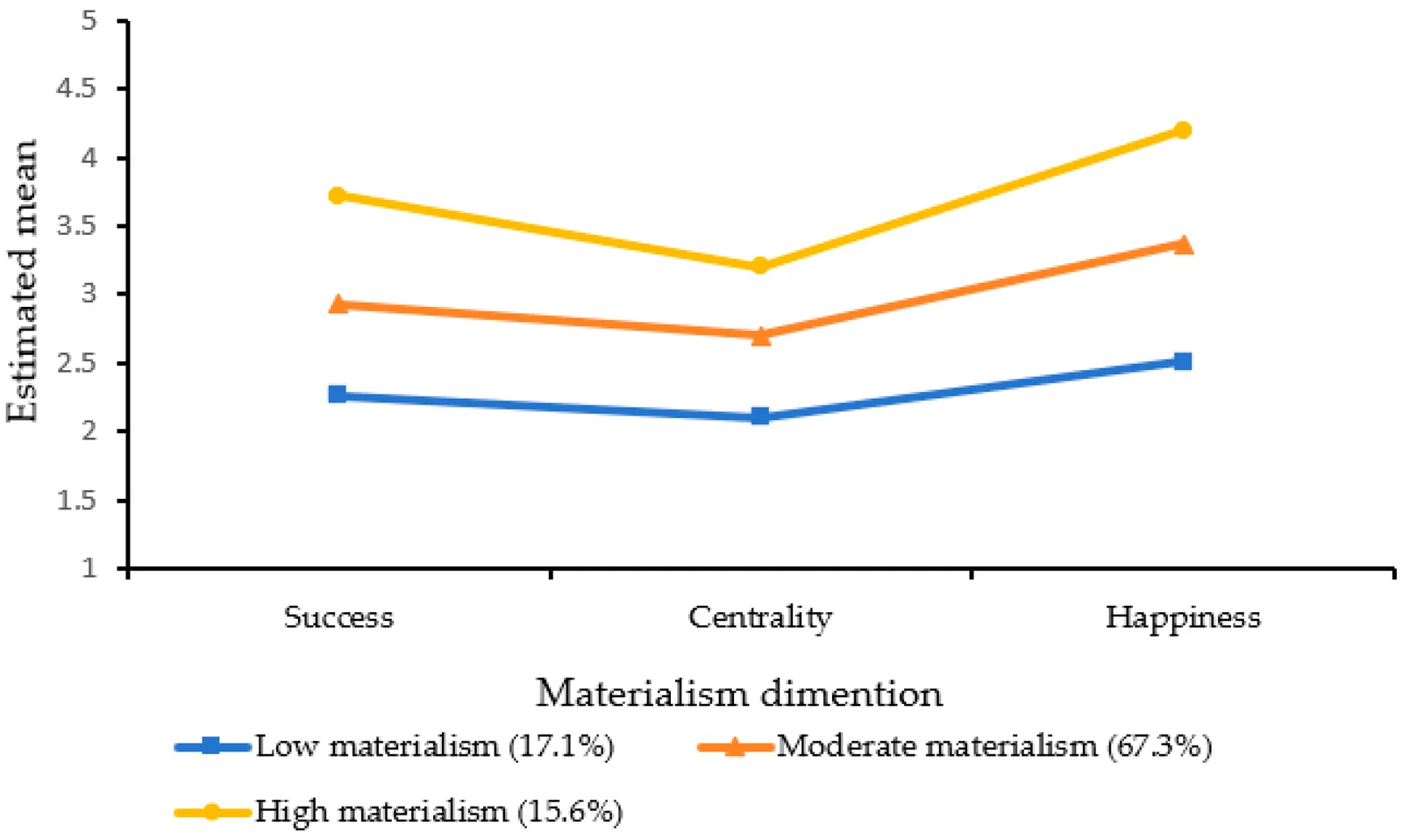
Estimated Means of the Three Materialism Dimensions Across the Low-, Moderate-, and High-Materialism Profiles. Note. Profile means are based on the retained MG4 multiple-group model, in which profile means were constrained to equality across SFES groups. The three materialism dimensions were scored on a 1–5 scale, with higher scores indicating greater materialism. Percentages in parentheses represent model-estimated profile proportions. SFES = subjective family economic status.

The retained solution comprised low-, moderate-, and high-materialism profiles (Figure 1). The three profiles primarily reflected quantitative differences in the overall level of materialism rather than qualitatively distinct profile shapes. Accordingly, the profile labels are used descriptively to denote relative materialism levels. Model-estimated proportions were 17.1%, 67.3%, and 15.6% for the low-, moderate-, and high-materialism profiles, respectively.

### 3.5. Adjusted T2 Well-Being Across Materialism Profiles and SFES Groups

All T2 distal-outcome estimates reported below were obtained using BCH models, which accounted for uncertainty in profile assignment and adjusted for the corresponding T1 outcome, gender, age, residence, and only-child status. Covariate-adjusted means for the nine combinations of SFES group and materialism profile are presented in Table 4.

The overall Wald test indicated significant variation across the nine combinations of SFES group and materialism profile for life satisfaction, *χ*^2^(8) = 28.84, *p* < 0.001, and for depressive symptoms, *χ*^2^(8) = 32.89, *p* < 0.001. To further examine these differences, planned high-versus-low materialism contrasts were conducted within each SFES group, as shown in Table 5.

Within each SFES group, adjusted life satisfaction was lower in the high-materialism profile than in the low-materialism profile, although none of the three planned contrasts remained statistically significant after Holm correction. In contrast, adjusted depressive symptoms were significantly higher in the high-materialism profile than in the low-materialism profile within all three SFES groups after Holm correction.

The interaction-type Wald tests were not significant for either life satisfaction, *χ*^2^(4) = 2.55, *p* = 0.636, or depressive symptoms, *χ*^2^(4) = 2.85, *p* = 0.584, providing no evidence that the magnitude of materialism-profile differences varied systematically across SFES levels. At the descriptive level, however, the difficult-SFES/high-materialism combination showed the lowest adjusted life satisfaction and the highest adjusted depressive symptoms among the nine groups.

### 3.6. Sensitivity and Complementary Analyses

#### 3.6.1. Negative Binomial Analysis of PHQ-9 Total Scores

Given the positive skew and substantial proportion of zero scores in the PHQ-9 distribution, the BCH analyses of depressive symptoms were repeated using the conventional 0–27 PHQ-9 total score under a negative binomial specification, with the same baseline and demographic adjustments as in the primary analysis. The overall difference across the nine combinations of SFES group and materialism profile remained significant, *χ*^2^(8) = 40.24, *p* < 0.001. The high-versus-low materialism rate ratios for T2 depressive symptoms were 1.73 (95% CI [1.08, 2.77], Holm-adjusted *p* = 0.022) in the difficult group, 1.91 (95% CI [1.23, 2.97], Holm-adjusted *p* = 0.008) in the below-average group, and 2.52 (95% CI [1.68, 3.78], Holm-adjusted *p* < 0.003) in the average-or-above group. The interaction-type test remained nonsignificant, *χ*^2^(4) = 2.08, *p* = 0.720. Thus, the negative binomial BCH analysis reproduced the primary depressive-symptom pattern: the high-materialism profile showed significantly higher depressive symptoms than the low-materialism profile within each SFES group, while there remained no evidence that the magnitude of these profile differences varied across SFES levels.

#### 3.6.2. Item-Level Materialism LPA

To evaluate whether the overall profile pattern depended on aggregating the 13 materialism items into three subscale scores, the LPA was repeated using all 13 individual items directly as indicators in the full analytic sample. The aLMR test supported the three-profile solution over the two-profile solution, whereas the four- and five-profile solutions were not supported relative to their respective preceding solutions. The retained three-profile solution showed acceptable classification accuracy, and its best log-likelihood was replicated. The corresponding fit statistics are presented in Table 6.

Although AIC, BIC, and aBIC continued to decrease with additional profiles, the four- and five-profile solutions each introduced a very small profile comprising approximately 3% of the sample. Inspection of the item means indicated that these small profiles appeared to be characterized primarily by discrepant response patterns between originally reverse-keyed and positively worded items rather than by a substantively distinct materialism configuration. For descriptive purposes, the item means within each profile were summarized separately for the Success, Centrality, and Happiness dimensions. The resulting dimension-level means for Success, Centrality, and Happiness were 2.34, 2.16, and 2.52 for the low-materialism profile; 2.86, 2.65, and 3.31 for the moderate-materialism profile; and 3.56, 3.10, and 4.04 for the high-materialism profile, respectively. Thus, a similar low-to-moderate-to-high pattern was reproduced when the individual items were analyzed directly, providing supplementary evidence that the overall profile pattern was not an artifact of aggregating the materialism items into subscale scores.

#### 3.6.3. Complementary Variable-Centered Analysis

The complementary variable-centered analyses yielded findings broadly consistent with the person-centered results. For T2 life satisfaction, the omnibus Materialism × SFES interaction was not significant, Wald *χ*^2^(2) = 0.94, *p* = 0.625. The estimated materialism slopes were negative in the difficult (*B* = −0.110, *SE* = 0.085, *p* = 0.196), below-average (*B* = −0.127, *SE* = 0.062, *p* = 0.038), and average-or-above (*B* = −0.187, *SE* = 0.050, *p* < 0.001) groups; *R*^2^ = 0.286. For T2 depressive symptoms, the omnibus interaction was also nonsignificant, Wald *χ*^2^(2) = 0.49, *p* = 0.785. The estimated materialism slopes were positive in the difficult (*B* = 0.091, *SE* = 0.040, *p* = 0.025), below-average (*B* = 0.087, *SE* = 0.029, *p* = 0.003), and average-or-above (*B* = 0.112, *SE* = 0.026, *p* < 0.001) groups; *R*^2^ = 0.254. Overall, the nonsignificant omnibus interactions for both outcomes provided no evidence that SFES moderated the associations between materialism and either T2 outcome. The conditional-slope pattern was directionally consistent with the profile-based results, with closer correspondence for depressive symptoms.

## 4. Discussion

The present study used a person-centered approach to examine materialism profiles across different levels of subjective family economic status (SFES) among Chinese higher vocational college students and their prospective associations with subjective well-being. Several key findings emerged.

First, low-, moderate-, and high-materialism profiles were identified within each of the three SFES groups. These profiles primarily reflected quantitative differences in the overall level of materialism rather than qualitatively distinct configurations across the Success, Centrality, and Happiness dimensions. This pattern suggests that heterogeneity in materialistic values among vocational college students may be expressed mainly in the degree to which materialistic values are endorsed, rather than in markedly different combinations of the three materialism dimensions. In other words, students differed primarily in how strongly they endorsed materialistic values overall, while the relative pattern across the three dimensions remained broadly similar. This level-based pattern was also reproduced in the item-level sensitivity analysis using all 13 materialism items as profile indicators.

Second, the materialism profile structure showed substantial similarity across the three SFES groups. Multiple-group comparisons supported a model in which profile means, indicator variances, and profile proportions were comparable across the difficult, below-average, and average-or-above SFES groups. This finding suggests that the basic pattern of heterogeneity in materialistic values was broadly consistent across different perceived family economic circumstances. That is, although students differed in their subjective family economic backgrounds, these differences did not appear to be accompanied by fundamentally different materialism profile structures. Rather, the low-, moderate-, and high-materialism profiles showed similar patterns and relative proportions across the three SFES groups.

Third, after adjusting for the corresponding T1 outcome and demographic covariates, both T2 life satisfaction and depressive symptoms showed significant overall variation across the nine combinations of SFES group and materialism profile. Further comparisons within each SFES group revealed different patterns for life satisfaction and depressive symptoms. For depressive symptoms, students in the high-materialism profile had significantly higher adjusted scores than those in the low-materialism profile within each SFES group. All three high-versus-low contrasts remained significant after Holm correction. The standardized high-versus-low differences in depressive symptoms ranged from 0.44 to 0.57 across the three SFES groups, suggesting that the differences were approximately moderate in magnitude. For life satisfaction, students in the high-materialism profile had lower adjusted scores than those in the low-materialism profile within each SFES group. However, none of the three contrasts remained significant after Holm correction. Thus, the association between materialism profiles and subsequent well-being was more consistently evident for depressive symptoms than for life satisfaction. One possible explanation for this difference is that depressive symptoms and life satisfaction capture distinct aspects of well-being. Depressive symptoms reflect psychological distress more directly (Kroenke et al., 2001), whereas life satisfaction represents a broader cognitive evaluation of one’s overall life circumstances (Diener et al., 1985) and may therefore be shaped by a wider range of personal and contextual factors. As a result, differences associated with materialistic values may be more readily reflected in depressive symptoms than in global evaluations of life satisfaction. This interpretation remains tentative; however, the differential associations of materialism with positive and negative aspects of well-being warrant further investigation.

Despite differences in the strength of the findings for the two well-being indicators, the overall direction of the profile differences was broadly consistent across SFES groups. Students in the high-materialism profile generally showed less favorable subsequent well-being than those in the low-materialism profile, with this pattern being particularly evident for depressive symptoms. This pattern is broadly consistent with previous longitudinal evidence linking stronger materialistic values to poorer subsequent well-being among Chinese university students (R. Wang et al., 2017). From the perspective of self-determination theory, materialism represents a value orientation that places considerable importance on externally defined indicators of success, such as wealth, possessions, and social status (Deci & Ryan, 2000; Ryan & Deci, 2017). When these external markers occupy a more central place in how individuals evaluate success and a desirable life, their sense of well-being may become more closely tied to the attainment of material and socially valued outcomes. Such an orientation has generally been associated with lower well-being and greater psychological distress (Dittmar et al., 2014; Kasser, 2016). This perspective may help explain why students in the high-materialism profiles showed a less favorable pattern of subsequent well-being than those in the low-materialism profiles.

The Wald tests of the SFES group × materialism profile interaction were not significant for either life satisfaction or depressive symptoms. This indicates that the association between materialism profiles and subsequent well-being did not differ significantly across levels of perceived family economic circumstances. In other words, the association between materialism profiles and well-being did not systematically strengthen or weaken across SFES levels. This finding is broadly consistent with previous meta-analytic evidence indicating that personal and household income did not significantly moderate the association between materialism and well-being (Dittmar et al., 2014), although objective income and subjective family economic status represent conceptually distinct indicators of economic circumstances. At the descriptive level, however, students in the difficult-SFES/high-materialism group showed the lowest adjusted life satisfaction and the highest adjusted depressive symptoms. From the perspective of scarcity theory, perceived limitations in economic resources may heighten the salience of material and financial concerns (Mullainathan & Shafir, 2013; Shah et al., 2012). For students who perceive their family economic circumstances as difficult while also placing strong importance on material attainment, the gap between what they value and the resources they perceive as available may become particularly salient, which may be associated with poorer psychological well-being. However, given the nonsignificant interaction tests, this descriptive pattern should not be interpreted as evidence that lower SFES strengthened the association between materialism and well-being.

The findings highlight the complementary value of person-centered and variable-centered approaches. The person-centered analyses revealed heterogeneity in materialistic values within each SFES group and allowed the similarity of profile structures to be examined across different perceived family economic circumstances. They also provided a way to compare the well-being of students characterized by different combinations of SFES and materialism profiles. In contrast, the complementary variable-centered analysis examined the average association between continuous materialism and well-being, as well as whether this association varied across levels of SFES. Notably, the variable-centered results were consistent with the person-centered findings in showing no significant moderation by SFES. Taken together, these approaches provide different but complementary perspectives: the person-centered approach captures heterogeneity among students, whereas the variable-centered approach summarizes associations at the overall sample level.

Several theoretical implications also emerge from these findings. First, the emergence of similar low-, moderate-, and high-materialism profiles across SFES groups suggests that individual differences in materialistic values may be expressed primarily in their overall level rather than in fundamentally different configurations of materialism across family economic circumstances. Second, the broadly consistent association between higher materialism and less favorable subsequent well-being across SFES groups, particularly for depressive symptoms, suggests that the psychological relevance of materialistic values is not confined to students from a particular economic background. This extends existing accounts of materialism by showing that its association with well-being can be observed across different levels of perceived family economic circumstances. At the same time, the absence of significant SFES moderation indicates that less favorable perceived family economic circumstances should not be assumed to systematically intensify the association between materialism and well-being. Together, these findings suggest that the relevance of materialistic values to psychological well-being extends across different family economic circumstances, rather than being confined to a particular level of SFES.

The findings may further inform efforts to support the well-being of vocational college students. Because higher materialism was generally associated with less favorable subsequent well-being across SFES groups, particularly with higher depressive symptoms, attention to materialistic values may be relevant across the range of perceived family economic circumstances rather than only among students who perceive their family circumstances as difficult. In educational and counseling settings, it may therefore be useful to encourage students to develop broader sources of personal value and life meaning that are not centered primarily on wealth, possessions, or social status. The more consistent differences observed for depressive symptoms further suggest that students with strong materialistic orientations may warrant particular attention in efforts to identify and support psychological distress. At the same time, students who perceive their family economic circumstances as difficult may benefit from support that acknowledges the pressures associated with financial concerns without assuming that less favorable perceived family economic circumstances necessarily amplify the psychological correlates of materialism. These implications should be viewed as directions for prevention and student support rather than evidence that changing materialistic values would itself improve well-being, which would require direct intervention research.

Several limitations should be considered when interpreting the present findings. First, although the two-wave prospective design and adjustment for the corresponding T1 well-being indicators strengthen the temporal interpretation of the observed associations, the study cannot establish causal effects of materialism or perceived family economic circumstances on subsequent well-being. Reverse or bidirectional associations remain possible, and the observed relationships may also reflect unmeasured factors such as personality characteristics, parenting experiences, peer influences, or social media use. In addition, self-determination theory and scarcity theory were used to provide possible interpretations of the findings, but the mechanisms proposed by these theoretical perspectives were not directly tested in the present analyses. Future longitudinal research that more explicitly examines temporal direction and tests the relevant theoretical mechanisms may provide a clearer understanding of these associations.

Second, several measurement-related limitations should be acknowledged. SFES was assessed using a single item with four response categories and therefore reflects students’ subjective evaluation of their family economic circumstances rather than a comprehensive measure of socioeconomic status. Although such subjective evaluations provide meaningful information about perceived economic conditions, the four-category response format may not fully capture more subtle variation in perceived family economic circumstances. Future research would benefit from combining subjective assessments with objective indicators such as household income, parental education, and parental occupation. In addition, the internal consistency of the three materialism subscales was modest. Supplementary split-half estimates, confirmatory factor analysis, and the item-level LPA using all 13 materialism items provided converging support for the measurement structure and the general low-to-high profile pattern; nevertheless, limited reliability may have reduced the precision with which individual differences in the three materialism dimensions were represented. Replication of the identified profiles in independent samples would further strengthen confidence in their stability. Finally, all focal variables were assessed using self-report measures, which may be susceptible to shared method variance and response biases. Future studies incorporating multiple informants, behavioral indicators, or other assessment methods could provide complementary evidence.

Third, attrition and sample composition may have affected the representativeness of the analytic sample. The T1-to-T2 retention rate was 42.7%, and participants retained at T2 differed from those not retained in age, gender, and grade, although differences in the principal psychological variables were generally small or nonsignificant. Selective attrition, therefore, cannot be completely ruled out. The analytic sample was also predominantly female, which may limit the extent to which the observed patterns generalize to male vocational college students. Future studies with more balanced samples and improved participant retention would help evaluate the robustness of these findings across demographic groups.

Finally, the generalizability of the findings should be considered with caution. Participants were recruited from a limited number of higher education institutions in Southwest China, and the analyses did not explicitly account for the nesting of students within institutions or classes. Potential non-independence arising from shared institutional or classroom environments was therefore not explicitly modeled. Moreover, because the study did not include students from general universities or other educational settings, the present findings should not be interpreted as uniquely characteristic of vocational college students. Nearly half of the analytic sample were left-behind students; although left-behind status was included in the descriptive, correlational, and attrition analyses, its potential role in the associations among family economic circumstances, materialism, and well-being was not examined in depth. Future research could directly compare vocational and general university students, include samples from different regions and cultural contexts, and examine whether family separation or other contextual characteristics modify the observed patterns.

## 5. Conclusions

In conclusion, the present study identified comparable low-, moderate-, and high-materialism profiles across different levels of subjective family economic status (SFES) among Chinese vocational college students. The profile structure and relative proportions were broadly similar across SFES groups, suggesting that heterogeneity in materialistic values was expressed primarily in overall level rather than in fundamentally different configurations across family economic circumstances. Higher materialism profiles were generally associated with less favorable subsequent well-being, with the pattern being more consistent for depressive symptoms than for life satisfaction. However, there was no evidence that the magnitude of materialism-profile differences in well-being varied systematically across SFES levels, a conclusion that was also supported by the complementary variable-centered analyses. Overall, these findings suggest that materialistic values are relevant to vocational college students’ well-being across different perceived family economic circumstances and highlight the relevance of materialism profiles for understanding subsequent well-being.

## Figures and Tables

**Table 1:** Descriptive Statistics and Correlations Among Study Variables.

**Variable**	***M***	***SD***	**1**	**2**	**3**	**4**	**5**	**6**	**7**	**8**	**9**
1. T1 Materialism	2.93	0.49	1.00								
2. T1 MAT–Success	2.94	0.59	0.85 **	1.00							
3. T1 MAT–Centrality	2.68	0.56	0.79 **	0.46 **	1.00						
4. T1 MAT–Happiness	3.35	0.70	0.76 **	0.53 **	0.41 **	1.00					
5. T1 SFES	1.23	0.77	0.03	−0.06 *	0.12 **	0.03	1.00				
6. T1 Life Satisfaction	3.25	0.82	−0.24 **	−0.32 **	−0.10 **	−0.14 **	0.36 **	1.00			
7. T1 Depressive Symptoms	0.40	0.42	0.22 **	0.23 **	0.18 **	0.08 **	−0.14 **	−0.36 **	1.00		
8. T2 Life Satisfaction	3.48	0.75	−0.19 **	−0.24 **	−0.09 **	−0.10 **	0.27 **	0.51 **	−0.32 **	1.00	
9. T2 Depressive Symptoms	0.33	0.39	0.19 **	0.24 **	0.12 **	0.09 **	−0.09 **	−0.24 **	0.48 **	−0.36 **	1.00
10. Age	18.68	0.87	−0.05 *	0.01	−0.10 **	−0.05	−0.11 **	−0.01	−0.02	−0.02	−0.02
11. Gender	0.70	0.46	0.21 **	0.09 **	0.25 **	0.17 **	0.01	0.00	−0.01	0.06 *	−0.10 **
12. Residence	0.66	0.48	−0.04	0.01	−0.08 **	−0.03	−0.25 **	−0.12 **	0.07 **	−0.12 **	0.00
13. Grade	1.29	0.49	0.01	0.05 *	−0.04	0.02	−0.04	0.02	−0.06 *	−0.03	−0.05 *
14. Only-Child Status	0.20	0.40	−0.06 **	−0.06 *	−0.07 **	−0.02	0.11 **	0.08 **	−0.03	0.05 *	0.00
15. Left-Behind Status	0.49	0.50	0.04	0.05 *	0.00	0.03	−0.10 **	−0.06 *	0.00	−0.03	0.01
**Variable**	**10**		**11**		**12**		**13**		**14**	**15**	
10. Age	1.00										
11. Gender	−0.06 *		1.00								
12. Residence	0.08 **		0.02		1.00						
13. Grade	0.57 **		0.08 **		0.09 **		1.00				
14. Only-Child Status	−0.01		−0.12 **		−0.22 **		0.00		1.00		
15. Left-Behind Status	0.03		0.00		0.18 **		0.01		−0.16 **	1.00	

Note. *M* = mean; *SD* = standard deviation; T1 = Time 1; T2 = Time 2; MAT = materialism; SFES = subjective family economic status. SFES reflects perceived family economic condition and was coded 0–3. Gender: 0 = male, 1 = female; residence: 0 = urban, 1 = rural; grade: 1 = first year, 2 = second year, 3 = third year; only-child status: 0 = no, 1 = yes; left-behind status: 0 = no, 1 = yes. * *p* < 0.05; ** *p* < 0.01.

**Table 2:** Fit Indices for Materialism LPAs Within the Three SFES Groups.

SFES Group	Profiles	LL	AIC	BIC	aBIC	Entropy	aLMR *p*	BLRT *p*	Smallest Profile
Difficult	1	−962.277	1936.555	1959.440	1940.407	—	—	—	—
	2	−888.918	1797.836	1835.977	1804.256	0.607	0.062	<0.001	41.8%
	**3**	**−856.691**	**1741.382**	**1794.780**	**1750.371**	**0.713**	**<0.001**	**<0.001**	**18.7%**
	4	−849.677	1735.353	1804.007	1746.910	0.745	0.055	0.030	1.7%
	5	−844.787	1733.575	1817.486	1747.699	0.749	0.156	0.667	1.5%
Below average	1	−1729.944	3471.887	3498.368	3479.319	—	—	—	—
	2	−1605.120	3230.241	3274.375	3242.627	0.579	0.013	<0.001	43.7%
	**3**	**−1542.495**	**3112.990**	**3174.779**	**3130.332**	**0.733**	**0.023**	**<0.001**	**15.8%**
	4	−1514.906	3065.812	3145.255	3088.108	0.739	0.044	<0.001	3.1%
	5	−1499.489	3042.978	3140.074	3070.229	0.784	0.043	<0.001	0.7%
Average or above	1	−1897.060	3806.121	3833.393	3814.342	—	—	—	—
	2	−1773.421	3566.841	3612.295	3580.543	0.532	0.088	<0.001	46.9%
	**3**	**−1704.705**	**3437.411**	**3501.046**	**3456.593**	**0.737**	**0.013**	**<0.001**	**11.7%**
	4	−1685.052	3406.104	3487.921	3430.767	0.726	0.031	<0.001	3.4%
	5	−1674.328	3392.655	3492.653	3422.799	0.723	0.007	<0.001	2.6%

Note. LPA = latent profile analysis; SFES = subjective family economic status; LL = log-likelihood; AIC = Akaike information criterion; BIC = Bayesian information criterion; aBIC = sample-size-adjusted BIC; aLMR = adjusted Lo–Mendell–Rubin likelihood ratio test; BLRT = bootstrap likelihood ratio test. “Smallest profile” denotes the smallest model-estimated profile proportion rather than the number of cases assigned by modal classification. Bold rows indicate the retained three-profile solutions.

**Table 3:** Comparison of Multiple-Group Materialism Profile Models Across SFES Groups.

Model	Cross-SFES Constraints	Parameters	LL	AIC	BIC	aBIC	CAIC	Entropy
MG1	None (configural)	44	−5836.808	11,761.616	11,999.351	11,859.570	12,043.351	0.865
MG2	Equal profile means	26	−5868.237	11,788.474	11,928.954	11,846.356	11,954.954	0.863
MG3	Equal profile means and variances	20	−5870.135	11,780.270	11,888.331	11,824.795	11,908.331	0.863
**MG4**	**Equal profile means, variances, and proportions**	**16**	**−5873.883**	**11,779.766**	**11,866.215**	**11,815.386**	**11,882.215**	**0.863**

Note. SFES = subjective family economic status; LL = log-likelihood; AIC = Akaike information criterion; BIC = Bayesian information criterion; aBIC = sample-size-adjusted BIC; CAIC = consistent Akaike information criterion; MG1 = configural model; MG2 = equal profile means across SFES groups; MG3 = equal profile means and indicator variances; MG4 = equal profile means, indicator variances, and profile proportions. The selected model is shown in bold.

**Table 4:** Adjusted T2 Well-Being Outcomes Across Materialism Profiles and SFES Groups.

SFES	Materialism Profile	Life Satisfaction *M (SE)*	Depressive Symptoms *M (SE)*
Difficult	Low	3.393 (0.114)	0.321 (0.041)
	Moderate	3.453 (0.054)	0.314 (0.027)
	High	3.191 (0.127)	0.492 (0.073)
Below average	Low	3.585 (0.092)	0.272 (0.041)
	Moderate	3.413 (0.037)	0.345 (0.020)
	High	3.363 (0.093)	0.448 (0.051)
Average or above	Low	3.670 (0.065)	0.214 (0.029)
	Moderate	3.568 (0.032)	0.308 (0.017)
	High	3.458 (0.084)	0.434 (0.046)

Note. Means are BCH estimates adjusted for the corresponding T1 outcome, gender, age, residence, and only-child status. T1 = Time 1; BCH = Bolck–Croon–Hagenaars; SFES = subjective family economic status; *M* = mean; *SE* = standard error.

**Table 5:** Planned High-versus-Low Materialism Contrasts Within Each SFES Group.

Outcome	SFES	Mean Difference (High − Low)	*SE*	95% CI	Standardized Mean Difference	Holm-Adjusted *p*
Life satisfaction	Difficult	−0.202	0.169	[−0.533, 0.129]	−0.27	0.231
	Below average	−0.222	0.132	[−0.481, 0.037]	−0.30	0.185
	Average or above	−0.211	0.106	[−0.419, −0.004]	−0.28	0.138
Depressive symptoms	Difficult	0.171	0.083	[0.008, 0.334]	0.44	0.040
	Below average	0.176	0.065	[0.048, 0.303]	0.45	0.014
	Average or above	0.220	0.055	[0.113, 0.328]	0.57	<0.001

Note. Contrasts are based on the adjusted BCH models. SFES = subjective family economic status; BCH = Bolck–Croon–Hagenaars; *SE* = standard error; CI = confidence interval. Standardized mean differences were calculated using the observed T2 standard deviation of the corresponding outcome. The 95% confidence intervals are unadjusted for multiple comparisons, whereas *p* values are Holm-adjusted for the three planned contrasts within each outcome.

**Table 6:** Sensitivity of LPA Using the 13 Individual Materialism Items.

Profiles	LL	AIC	BIC	aBIC	Entropy	aLMR *p*	BLRT *p*	Smallest Profile
1	−29,662.907	59,377.814	59,518.293	59,435.696	—	—	—	—
2	−28,544.832	57,169.663	57,385.786	57,258.712	0.742	<0.001	<0.001	44.9%
**3**	**−28,175.666**	**56,459.331**	**56,751.097**	**56,579.548**	**0.771**	**0.002**	**<0.001**	**18.4%**
4	−27,900.955	55,937.910	56,305.318	56,089.293	0.816	0.340	<0.001	2.85%
5	−27,708.552	55,581.105	56,024.156	55,763.655	0.776	0.146	<0.001	2.59%

Note. LPA = latent profile analysis; LL = log-likelihood; AIC = Akaike information criterion; BIC = Bayesian information criterion; aBIC = sample-size-adjusted BIC; aLMR = adjusted Lo–Mendell–Rubin likelihood ratio test; BLRT = bootstrap likelihood ratio test. “Smallest profile” denotes the smallest model-estimated profile proportion rather than the number of cases assigned by modal classification. The retained three-profile solution is shown in bold.

## Data Availability

The data that support the findings of this study are available on reasonable request from the corresponding author. The data are not publicly available due to privacy or ethical restrictions.

## Abbreviations

The following abbreviations are used in this manuscript:

SFES	subjective family economic status
MAT	materialism
SDT	self-determination theory
